# Statistical Modeling of Lower Limb Kinetics During Deep Squat and Forward Lunge

**DOI:** 10.3389/fbioe.2020.00233

**Published:** 2020-04-02

**Authors:** Joris De Roeck, J. Van Houcke, D. Almeida, P. Galibarov, L. De Roeck, Emmanuel A. Audenaert

**Affiliations:** ^1^Department of Human Structure and Repair, Ghent University, Ghent, Belgium; ^2^Centre for Rapid and Sustainable Product Development, Polytechnic Institute of Leiria, Leiria, Portugal; ^3^AnyBody Technology A/S, Aalborg, Denmark; ^4^Department of Orthopaedic Surgery and Traumatology, Ghent University Hospital, Ghent, Belgium; ^5^Department of Trauma and Orthopaedics, Addenbrooke’s Hospital, Cambridge University Hospitals NHS Foundation Trust, Cambridge, United Kingdom; ^6^Department of Electromechanics, Op3Mech Research Group, University of Antwerp, Antwerp, Belgium

**Keywords:** lower limb kinetics, inverse dynamics, principal component analysis, musculoskeletal model, validation analysis

## Abstract

**Purpose:**

Modern statistics and higher computational power have opened novel possibilities to complex data analysis. While gait has been the utmost described motion in quantitative human motion analysis, descriptions of more challenging movements like the squat or lunge are currently lacking in the literature. The hip and knee joints are exposed to high forces and cause high morbidity and costs. Pre-surgical kinetic data acquisition on a patient-specific anatomy is also scarce in the literature. Studying the normal inter-patient kinetic variability may lead to other comparable studies to initiate more personalized therapies within the orthopedics.

**Methods:**

Trials are performed by 50 healthy young males who were not overweight and approximately of the same age and activity level. Spatial marker trajectories and ground reaction force registrations are imported into the Anybody Modeling System based on subject-specific geometry and the state-of-the-art TLEM 2.0 dataset. Hip and knee joint reaction forces were obtained by a simulation with an inverse dynamics approach. With these forces, a statistical model that accounts for inter-subject variability was created. For this, we applied a principal component analysis in order to enable variance decomposition. This way, noise can be rejected and we still contemplate all waveform data, instead of using deduced spatiotemporal parameters like peak flexion or stride length as done in many gait analyses. In addition, this current paper is, to the authors’ knowledge, the first to investigate the generalization of a kinetic model data toward the population.

**Results:**

Average knee reaction forces range up to 7.16 times body weight for the forwarded leg during lunge. Conversely, during squat, the load is evenly distributed. For both motions, a reliable and compact statistical model was created. In the lunge model, the first 12 modes accounts for 95.26% of inter-individual population variance. For the maximal-depth squat, this was 95.69% for the first 14 modes. Model accuracies will increase when including more principal components.

**Conclusion:**

Our model design was proved to be compact, accurate, and reliable. For models aimed at populations covering descriptive studies, the sample size must be at least 50.

## Introduction

In biomechanics, the safety and efficiency of novel surgical techniques as well as the development of biocompatible products ultimately rely on its capability of being tested on humans through clinical trials. The complete development chain of a new surgical technique or implant and their introduction into clinic practice is both time-consuming and economically demanding. Next to it, it is known that patient-specific surgery planning or implant design can improve the long-time outcome of an implant (Pietsch et al., 2013; Spencer-Gardner et al., 2016). This fact is due to the high anatomical variability between individuals and the different functional activities, which have a significant effect in the ratio of the force components on the lower limb between subjects (Kutzner et al., 2010) and on the functional alignment of the prosthetic components of a lower limb implant (Smoger, 2016; Spencer-Gardner et al., 2016). Within this context, methodologies such as statistical models of the human anatomy as well as kinematics or kinetics that account for the anatomical inter-variability of the population combined with biomechanical simulation studies can provide non-invasive pre-surgical clinical output.

Lower limb kinetics can be estimated based on musculoskeletal models and ground force plates using inverse dynamics (Carbone et al., 2012; Galloway et al., 2012; Vaitkus and Várady, 2015; Bagwell et al., 2016). These techniques do not often account for patient-specific variability as they use scaled generic models (Worsley et al., 2011; Vaitkus and Várady, 2015), while it was already widely shown that the geometry of the musculoskeletal models is very sensitive to muscle force predictions (Carbone et al., 2012). In addition, and to the authors knowledge, the available studies merely consider very limited population samples which may not be representative of the total variability of the lower limb anatomy. Lastly, the available literature lacks completeness as, to date, no study has considered a statistical model of the full lower limb, namely, on demanding tasks such as the deep squat and the forward lunge.

Hence, in order to create the foundations for the development and optimization of the design or the durability of orthopedic implants, it is mandatory to generate appropriate loading conditions that represent inter-patient variability across the population (Honari and Taylor, 2013; Bischoff et al., 2014). Patient-specific finite element analyses are the state-of-the-art technique to infer quantitative information on a specific design or performance of an arthroscopic implant (Shu et al., 2018). Taylor et al. (2012) found most studies to be focusing on variations on the morphological and bone properties rather than the consequences of variability because of loading. Furthermore, it has been proved that the application of single-representative models can be extended to account for variability by either parametrically or probabilistically varying the loading/boundary conditions. These approaches allow model generation which can significantly extend the coverage of the anatomical variability and ultimately create a powerful tool to assess the performance of medical devices (Taylor et al., 2012).

Recent developments in medical imaging significantly increased the accuracy of the three-dimension computational anatomical representation, enhancing the anatomical differences within a determined population (Almeida et al., 2016; Audenaert et al., 2019). Hence, combining the use of magnetic resonance imaging (MRI) with musculoskeletal models will provide us an insight on lower limb kinematics on patient-specific anatomies. The statistical analysis of kinematic time series by means of dimensionality reduction techniques such as principal component analysis (PCA) or independent component analysis is not novel *per se* (Daffertshofer et al., 2004; Galloway et al., 2012), but the inclusion of patient-specific anatomies is believed to more accurately represent inter-patient kinetic variability. Such approach, hereby presented, will allow for a large population of kinetic data to be generated without the time and the expense of collecting the motion capture data of hundreds of patients. Simultaneously, it will open the door to the generation of large simulated populations for use in clinical outcome simulation studies, injury biomechanics, musculoskeletal disease models, or implant design optimization (Henak et al., 2013; Zhang et al., 2016).

While the gait cycle has been the most researched activity in the current literature, it is not particularly demanding for the lower limb joints. For the purpose of implant wear testing, implant fixation, and joint stability analysis, there are other more challenging activities commonly performed in daily living that might be of particular interest (Hartmann et al., 2013). Clinical, experimental, and computational studies have clearly reported increased complication risk and wear rate under high contact stress conditions (Kang et al., 2008; O’Brien et al., 2015; de Ruiter et al., 2017).

In sum, the purpose of this study is to build statistical models of deep squatting and forward lunging for applications in pre-clinical testing of orthopedic implants and surgery in an asymptomatic adult population and ultimately to analyze and validate the inter-individual variations in lower limb kinetics.

## Materials and Methods

### Participants

Fifty-three asymptomatic volunteers participated in the study. In order to eliminate sex and race differences and reduce the potential influence of age and body mass index (BMI), only healthy Caucasian men who were not overweight and aged between 17 and 25 years are included. The admission requirement is practicing sports for at least 2 h a week. The subjects were asked to perform five times a smooth maximal-depth squat and a right forward lunge step with a predetermined frequency and fluency after a short training. In addition, the volunteers underwent full lower limbs MRI. An ethics committee (Ghent University Hospital, Belgium) approved these investigations (EC2014/0286). The characteristics of our study population are listed in Table 1. Because of missing data, there was no complete data acquisition for the squat among the three subjects.

**TABLE 1:** Demographic and anthropometric characteristics of the study population.

Demographic descriptor	Mean (95% CI*)	Normal values
Height (cm)	181.79 (180.08–183.51)	Not applicable
Weight (kg)	71.75 (69.63–73.88)	Not applicable
Body mass index (kg/m^2^)	21.70 (21.16–22.23)	18.5–25 (Waxman, 2004)
Sport activity (hours a week)	3.40 (2.76–4.03)	Not applicable
Center-edge angle (°)	28.41 (27.19–29.63)	25–39° (Audenaert et al., 2012; Ghaffari et al., 2018)
Alpha angle (°)	64.61 (62.38–66.84)	<55° (Audenaert et al., 2012; Ghaffari et al., 2018)
Centrum-collum-diaphyseal angle or neck-shaft angle (°)	129.24 (127.99–130.49)	125–135° (Audenaert et al., 2012)
Femoral anteversion angle (°)	9.40 (7.30–11.49)	<15° (Audenaert et al., 2012)

*Confidence interval of the mean.

In both examinations, 28 reflective markers are stuck on the skin on palpable anatomical landmarks. The application of skin markers to investigate kinetics is obvious but rather inaccurate. By contrast, using more accurate measurements with implants would raise ethical concerns.

### Instrumentation

Our motion capture acquisition strategy was based on a similar study by Deluzio et al. (Deluzio and Astephen, 2007). Spatial marker trajectory data and the corresponding force registrations are imported into the Anybody Modeling System (AMS version 7.1.0, Anybody Technology, Aalborg, Denmark) (Damsgaard et al., 2006) as well as geometric data from a 3-Tesla MAGNETOM Trio-Tim System MRI device (Siemens AG, Erlangen, Germany).

### Musculoskeletal Modeling

**FIGURE 1:**
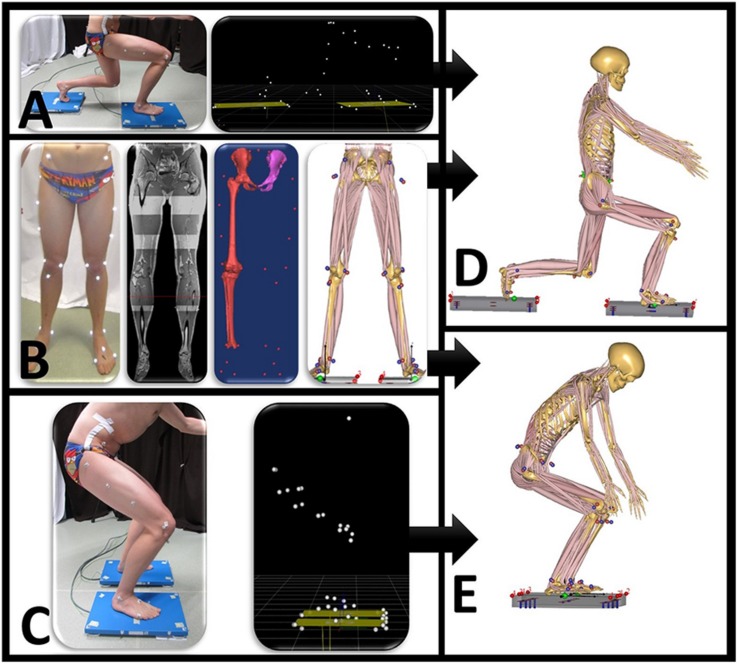
Overview of data input for the motion capture musculoskeletal simulation model. **(A)** Motion is performed when standing on two force plates. Motion capture data synchronized with ground reaction forces are exported as .c3d file. **(B)** Twenty-eight reflective markers are placed on anatomical bony landmarks. A MRI scan of the full lower limb is performed. Segmentation of pelvis, thigh, and shank with corresponding positions of marker landscape. **(C)** Motion capture squat model. Anybody squat **(D)** and lunge **(E)** model.

Motion capture musculoskeletal models were personalized with subject-specific bone geometry that was incorporated in a simulation model from the Twente Lower Extremity Model (TLEM 2.0) dataset (Carbone et al., 2015). An overview of the musculoskeletal model input is presented in Figure 1. In the simulation output, the forces are described in three fixed perpendicular planes.

### Data Processing

The output data from musculoskeletal models are numerous, multivariate, and multidimensional (Deluzio and Astephen, 2007; Lai et al., 2009). In contrast to some gait studies that modeled kinematic and kinetic data together, we used only kinetic data (Deluzio and Astephen, 2007; Reid et al., 2010; Galloway et al., 2012). We think that integrating linear quantities (forces) and rotation quantities (angles) is like comparing apples and oranges. On top of that, the kinetic data in Anybody is generated by an inverse dynamics approach starting from the kinematic data.

The beginning and end frames of all motion lab recordings are not useful due to irrelevant transients. Analogously, the peak evolution will vary from the center of the recorded data. Hence, data alignment and trimming are essential prior to incorporating the subjects’ motion recordings into a statistical model. These operations are executed using standard implementations in MATLAB (MathWorks, Natick, MA, United States).

The frame recorded with the peak knee flexion angle is defined as 50% progress of the motion. Trimming is based on knee flexion. For the lunge, the best is to consider only the closed chain part. As such, recordings where the right foot is not on the right force plate are left aside. Several arbitrary ways to execute an open-chain motion could be an important source of noise η. Noise is defined as artifacts when processing the input data to the output data (Lai et al., 2009). On top of data, we used only information from the leg that was the most loaded. So, in contrast to the squat data, a lot of waveform data are not used for the lunge.

Interpolation is performed to ensure that the measurements are running synchronized in real time. All trimmed measurements are subdivided into 0–50–100 proceedings, corresponding to the onset, the middle, and the finish of motion, respectively. Each set of kinetic data is arranged in a feature vector and concatenated into a training matrix. The training data matrix *X* contains observations in the rows and subjects in the columns as described in Eqs. [1] to [3].

(1) $$X=\left[x_{1},x_{2},x_{3},\ldots ,x_{i},\ldots ,x_{p-1},x_{p}\right]$$

An observation expresses several dynamic parameters on a certain progress of the aligned lunge or squat motion from 0 to 100%. For each participant *i*, the kinetic model input data are taken from the musculoskeletal model output. The kinetic variables are implemented into a subject vector *x*_*i*_ for the *i*th subject (out of *p*).*p* represents the number of training samples, being 53 for the lunge and 50 for the squat.

(2) $$x_{i}={\left[HJRF_{x},HJRF_{y},HJRF_{z},KJRF_{x},KJRF_{y},KJRF_{z},\right]}^{T}$$

The input matrices *JRF*_*axis*_ consist all of 101 observations *o*.

(3) J⁢R⁢Faxis=[measurement⁢ 1measurement⁢ 2……measurement⁢51⁢(at⁢maximal⁢right⁢knee⁢flexion)…measurement⁢101]

*D* serves as a diagonal matrix with row-wise standard deviations *d*_*o*_ for each observation *o*. The total number of observations is the multiplication of the number of dynamic variables and aligned time instances.

(4) $$D=\left[\begin{array}{cc} \begin{array}{cc} d_{1} & 0\\ 0 & d_{2} \end{array} & \begin{array}{cc} 0 & \ldots \\ 0 & 0 \end{array}\\ \begin{array}{cc} 0 & 0\\ \ldots & 0 \end{array} & \begin{array}{cc} \ldots & 0\\ 0 & d_{606} \end{array} \end{array}\right]$$

After normalization by row-wise standard deviation in [4] and [5] as well as mean centering in [6], a residual matrix *R* is created. *R* comprises the entry data for the model *M* as a measure of dispersion.

(5) $$\tilde{X}=\left[\begin{array}{ccc} \ldots & \begin{array}{c} \ldots \\ {\tilde{x}}_{i}\\ \ldots \end{array} & \ldots \end{array}\right]=D^{-1}\,X$$

(6) $$R=\left[\begin{array}{ccc} \ldots & \begin{array}{c} \ldots \\ {\hat{x}}_{i}\\ \ldots \end{array} & \ldots \end{array}\right]\,w\,i\,t\,h\,{\hat{x}}_{i}={\tilde{x}}_{i}-\bar{x}$$

PCA is a powerful dimensionality reduction technique developed by Karl Pearson. It is not a method to investigate the center size of the data but the common variability. PCA is mathematically defined as an orthogonal linear transformation. PCA transforms the data; as such, most of the variance of the data will come to lie in the first components. This allows us to create statistical models. Altogether PCA is a reliable tool in capturing the salient features of waveform data (Robbins et al., 2013; Jolliffe and Cadima, 2016).

Using this for a statistical model, it enables to generate population data from a small set of clinical data. The kinetic model should represent waveform data as a linear combination of vectors, representing the primary modes of variation in experimental data (Jolliffe et al., 2002; Saliba et al., 2018). Eigenvalues and eigenvectors have been created by singular value decomposition.

(7) $$R=U\,x\,L\,x\,A^{T}$$

In Eq. [7], *U* and *A* are the left and right singular vectors, so *U^T^*.*U* = *I* and *A^T^*.*A* = *I* because of orthogonality. *I* refers to the unity matrix. *L* is a diagonal matrix that contains the square roots of the eigenvalues $\sqrt{\lambda_{k}}$ belonging to *R^T^ x R*, as *k*{1,2,3,…,*p*}. *A^T^* contains the eigenvectors of *R^T^xR*, whereas *U* has the scaled versions of the principal component (PC) scores *u*_*ok*_. Here, *o* corresponds to the observation and *k* to the PC number. The PC scores are mentioned in Eq. [8].

(8) $$z_{o\,k}=u_{o\,k}\,\sqrt{\lambda_{k}}$$

The PC scores from a single waveform quantify the contribution of each feature. The variance of the scores for the *k*th eigenvalue of *R^T^ x R* amounts to $\frac{\lambda_{k}}{n-1}$, as λ_*k*_ represents the variance of the k*t*h PC, whereas *n* is the number of observations.

The cumulative variance of each mode *k* is defined as

(9) $$\text{Compactness}\,\left(M\right)=\frac{1}{p-1}\,\sum_{m=1}^{k}\lambda_{m}$$

The PC weight matrix *W* in Eq. [10] involves the correlation coefficients between components and test subject data.

(10) $$W=L\,x\,A^{T}$$

A set of patient data can be approximately reconstructed by using *t* selected PCs in Eq. [11].

(11) $${\hat{x}}_{i\,j}=D\,\sqrt{p-1}\,\sum_{i=1}^{t}u_{o\,i}\,\sqrt{l_{i}}\,a_{k\,i}+\bar{x}$$

As mentioned before, *D*represents the diagonal matrix of row-wise standard deviations and *p* stands for the subject count. $\sqrt{l_{i}}$ is the *i*th diagonal element of *L*, also from the singular value decomposition (Jolliffe et al., 2002; Galloway et al., 2012; Jolliffe and Cadima, 2016).

### Model Validation

Validation is defined as the process of ensuring that the dimensionality-reduced PCA model accurately represents real-world kinetics. Probably the most important problem arising with this process is the choice of the optimal number of the principal components to be retained. PCA projects the input data from a high dimensional space into a subspace of lower dimension, which can then further be divided into two separate subspaces: the *kinetic data subspace*, preserving the essence of the original kinetic data as lossless as possible, and the *noise subspace*, corresponding to the remaining tail of principal components associated with the smallest eigenvalues. Given the complexity of the problem of optimally defining the threshold between signal and noise principal components, the literature on the topic is overwhelming and beyond the scope of this work. Reliable results in distinguishing components that express meaningful correlations among variables as opposed to trivial components, explaining noise, have been provided using the Monte Carlo permutation test (Peres-Neto et al., 2005). The principal components were tested for representing valid correlations as opposed to residual error using the following two criteria: rank of roots and equality of roots (Jackson, 2005; Vasco, 2012).

Further, four quantitative model parameters are investigated. “Goodness” measures are chosen according to the statistical shape modeling study of Styner et al. (2003) in which there is also a PCA dimensionality reduction algorithm. This study is, to the authors’ best knowledge, the first to provide such an approach, implemented for a kinetic model.

#### Model Accuracy

(12) $$\text{Accuracy}\,\left(M\right)=\frac{1}{p}\,\sum_{i=1}^{p}{||{\hat{x}}_{i}\,\left(M\right)-x_{i}||}^{2}$$

The first validation test that analyzes relevant information is retained by the model or otherwise states how well the original data can be reconstructed from the model given the number of principal components retained. Here, the root-mean-square error (RMSE) is computed in Eq. [12] as the average absolute difference between the original training data and the reconstructed data for models with 80, 90, 95, and 98% variance of the original data.

#### Model Compactness

The model will be compact enough if it can describe the variance in kinetic measurements with a minimal number of modes. Eq. [9] is used to describe the compactness with the cumulative variance for a certain number of modes.

#### Model Generalization

(13) $$\text{Generalization}\,\left(M\right)=\frac{1}{T_{g}}\,\sum_{i=1}^{T_{g}}{||{\hat{x}}_{i}\,\left(M_{i}^{\prime }\right)-x_{i}||}^{2}$$

The model generalization quantifies the ability of models to represent new instances. The generalization ability is evaluated by performing a series of leave-one-out tests on the training data. The question here is: how many training samples are necessary to approach the population precisely? The generalization ability is therefore a means for *post hoc* sample size evaluation. If having enough training samples, we expect the model to be able to describe unseen data quite accurately (Wang and Shi, 2017). The generalization value can be interpreted as the median out-of-sample accuracy value.

The generalization evolution gives the RMSE between the excluded subject data and the best-matched 95% variance model *M*’ values of randomly selected training data by ascending number of training samples in the model *M*’. The higher the *T*_*g*_ test value in Eq. [13], the higher the precision of the median generalization value. Here the number of models created for each number of training samples amounts to *T*_*g*_ = 10,000.

#### Model Specificity

(14) $$\text{Specificity}\,\left(M\right)=\frac{1}{T_{s}}\,\sum_{i=1}^{T_{s}}{||{\hat{x}}_{i}^{\prime }\,\left(M\right)-x_{i}||}^{2}$$

A population model is able to generate new data. The model specificity measures the soundness of new instances randomly generated by the developed model *M*. Models with 80, 90, 95, and 98% of variance are tested. ${\hat{x}}_{i}^{\prime }\,\left(M\right)$ refers to a randomly generated subject.

We assume that the PCs of the model are normally distributed (Jolliffe et al., 2002; Jackson, 2005; Galloway et al., 2012). The specificity estimator is defined in Eq. [14]. For each observation *o*, an imaginary subject *i* is defined by choosing random normal distributed values *n* ∈ *N*(0,1) for each mode *m* in the model *M* as in Eq. [15].

(15) $${\hat{x}}_{i,o}^{\prime }\,\left(M\right)={\bar{x}}_{o}+d_{o}\,\sum_{m=1}^{p}n.\sqrt{\lambda_{m}}.z_{m}=\bar{x}+d_{o}\,\sum_{m=1}^{p}n.u_{m}$$

The RMSE is defined as the error between the virtually subject data and the most similar sample in the training dataset. The specificity value can be interpreted as the median approximation error of *T*_*s*_ generated subjects. The higher the *T*_*s*_ test value, the higher the precision of the specificity. Here the number of models created is set to *T*_*s*_ = 1,000,000.

## Results

The average hip and knee peak flexion angles are, respectively 95° and 104° for the lunge and 107° and 112° for the squat motion, respectively. The average peak hip joint reaction force (HJRF) amounts to 3.08 times body weight (BW) for the maximum-depth squat and 4.76 BW for lunging. The means for peak knee joint reaction force (KJRF) are still higher: 4.52 BW for squat motion and 7.16 BW for the lunge. The trimmed original waveform data from HJRF and KJRF of our musculoskeletal model are represented by gray curves in Figures 3–8.

**FIGURE 2:**
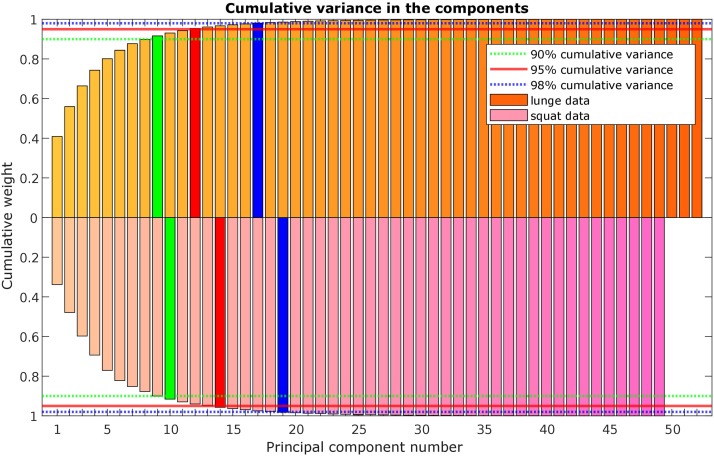
Scree plot with the cumulative variance of the modes (or principal components) in the lunge (orange) and squat (purple) kinetic model.

**FIGURE 3:**
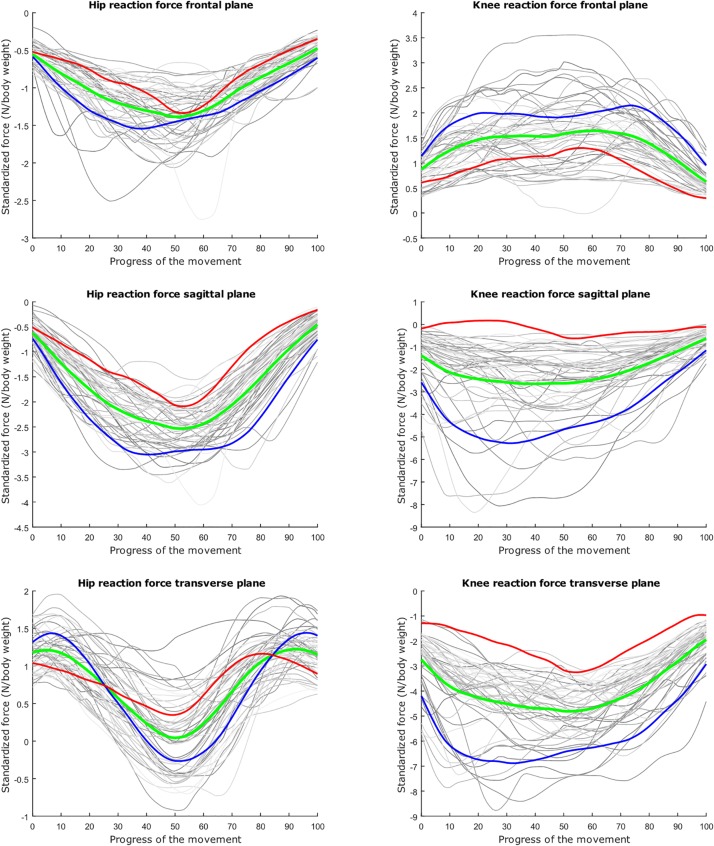
Relation between the kinetic waveform simulation output and the squat progress for each individual sample in gray. Mean values of the measurements in green ±2 standard deviations of the first mode in red and blue. The first mode accounts for 33.80% of the inter-subject population variance. Note the different *y* axis calibrations.

**FIGURE 4:**
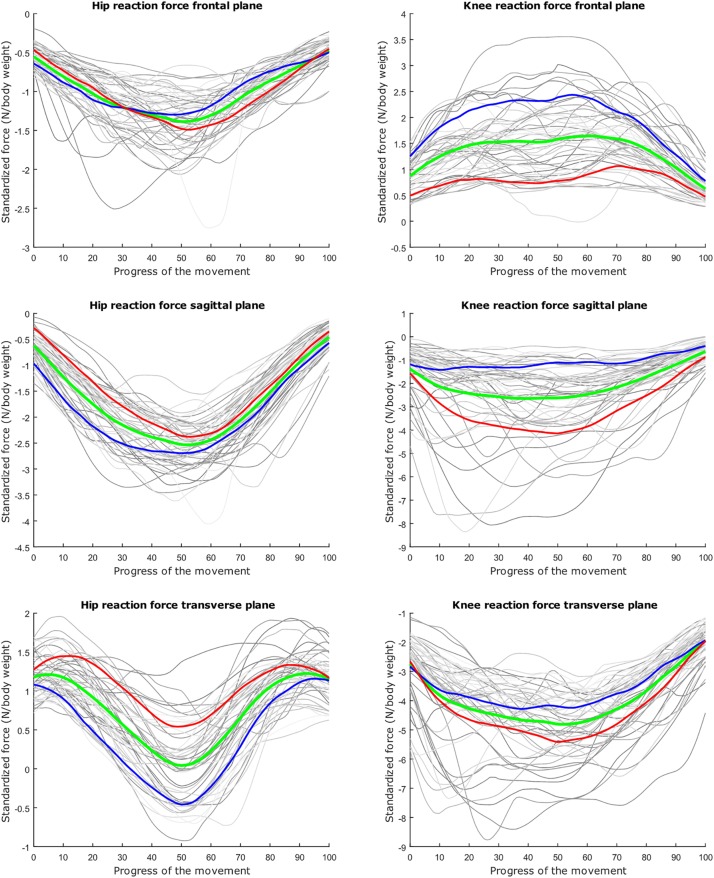
Mean values of joint reaction forces during deep squatting in green ±2 standard deviations of the second mode in red and blue. The second mode accounts for 14.05% of the inter-subject population variance.

**FIGURE 5:**
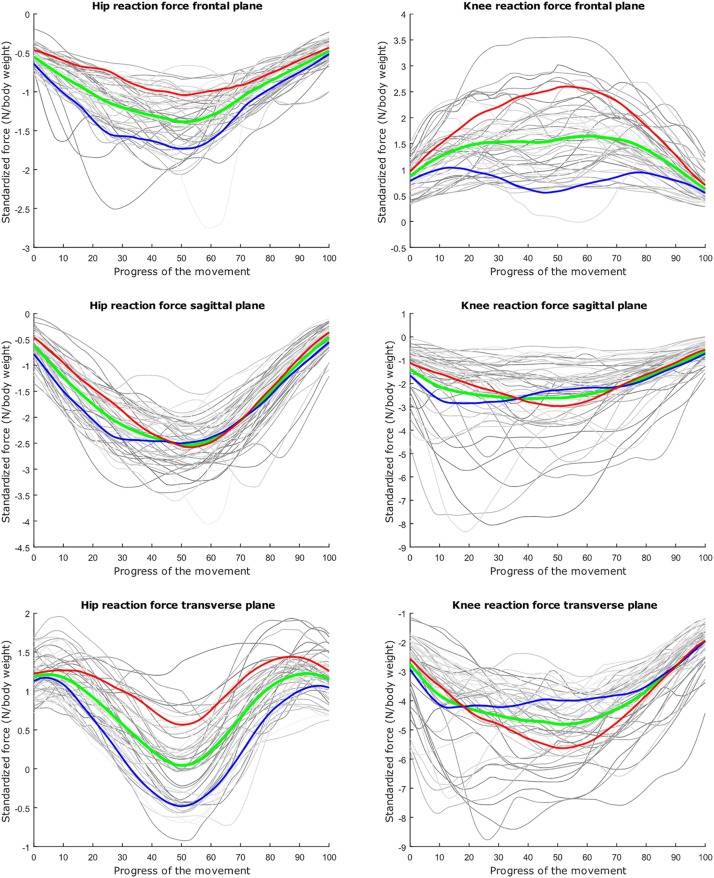
Mean values of joint reaction forces during deep squatting in green ±2 standard deviations of the third mode in red and blue. The third mode accounts for 11.88% of the inter-subject population variance.

**FIGURE 6:**
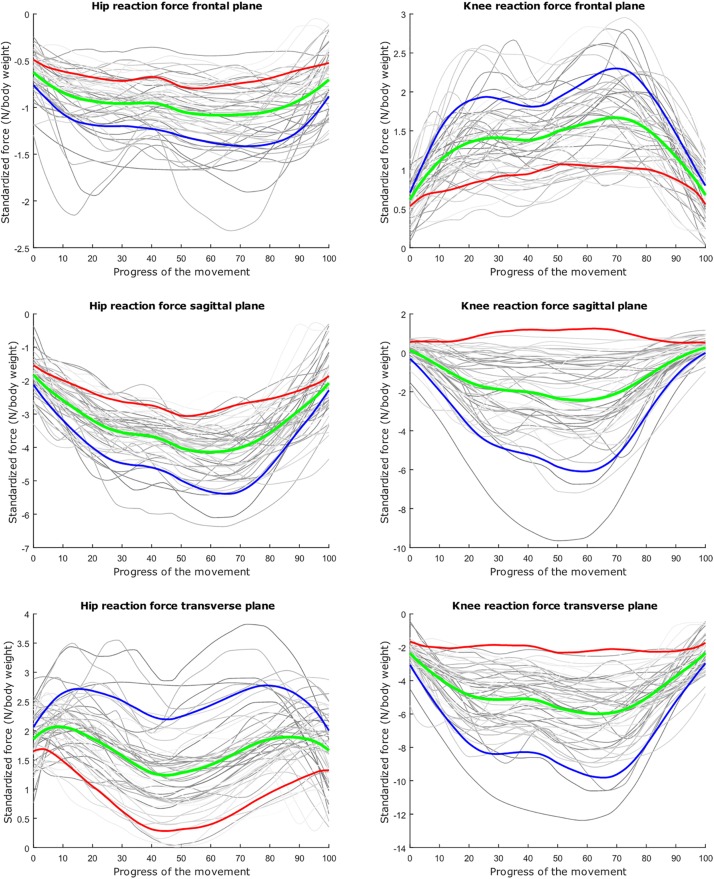
Mean values of joint reaction forces during lunging in green ±2 standard deviations of the first mode in red and blue. The first mode accounts for 40.87% of the inter-subject population variance.

**FIGURE 7:**
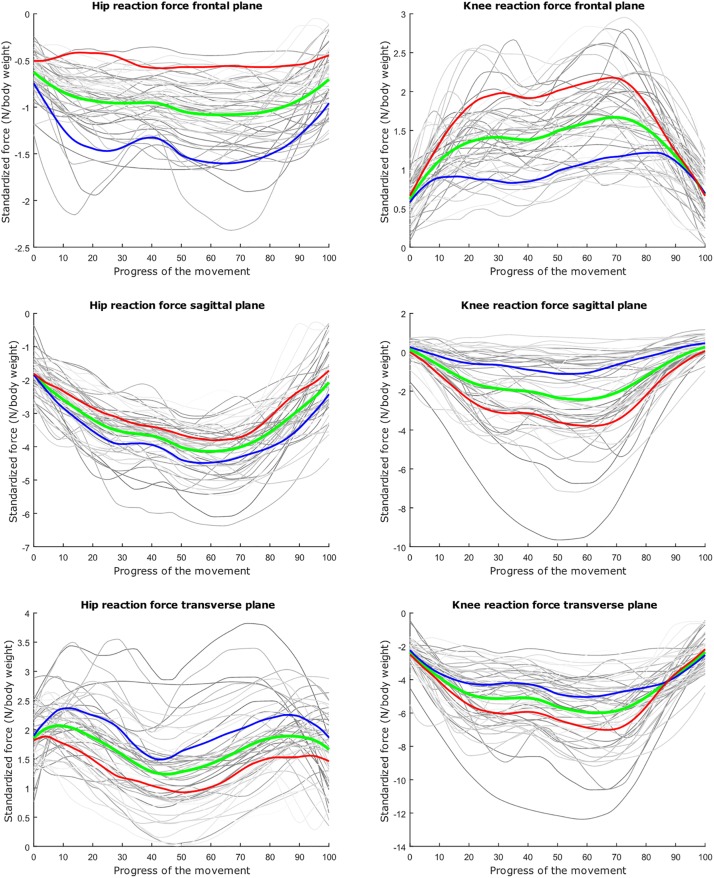
Mean values of joint reaction forces during lunging in green ±2 standard deviations of the second mode in red and blue. The second mode accounts for 15.07% of the inter-subject population variance.

**FIGURE 8:**
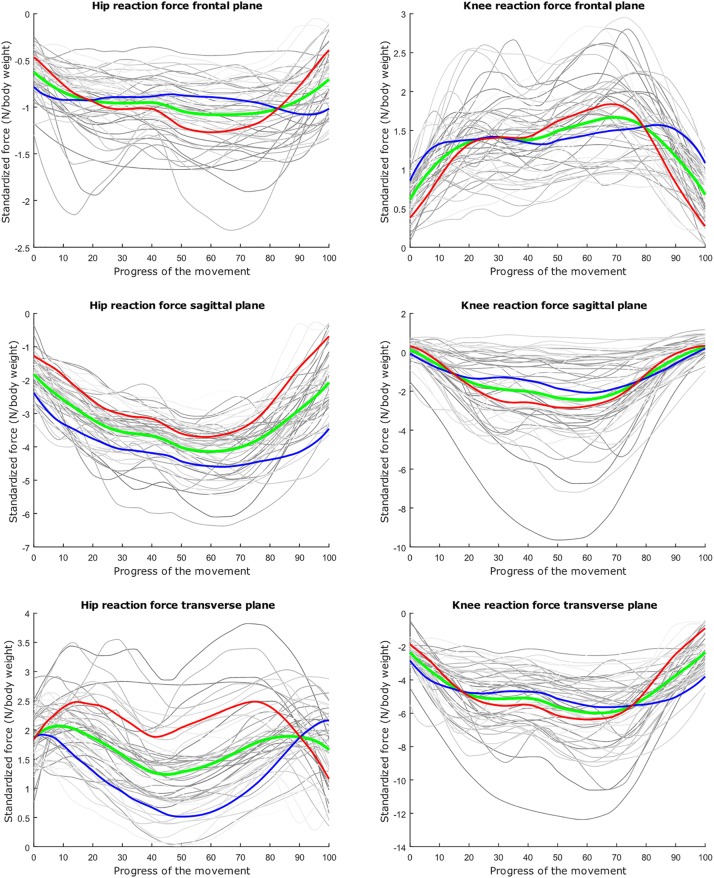
Mean values of joint reaction forces during lunging in green ±2 standard deviations of the third mode in red and blue. The third mode accounts for 10.46% of the inter-subject population variance.

**FIGURE 9:**
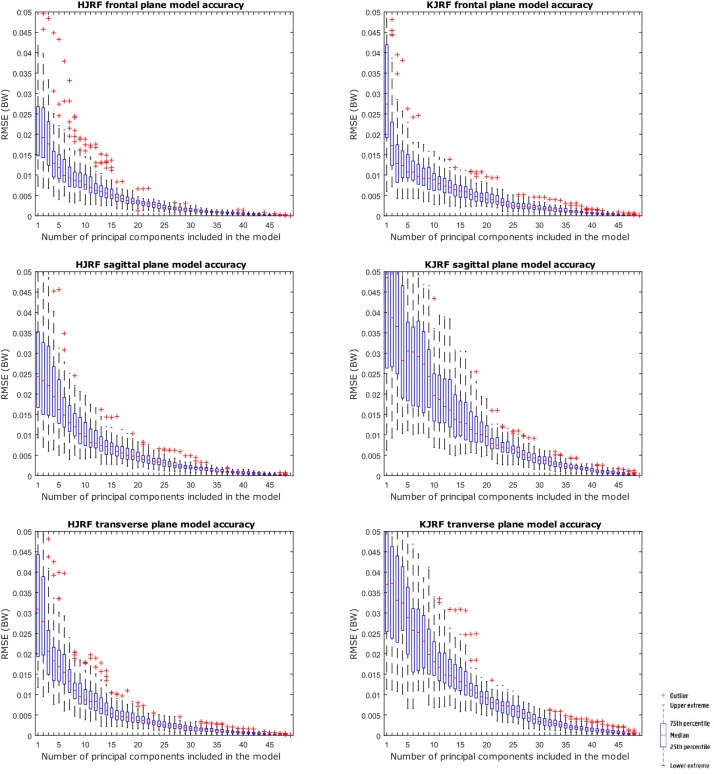
RMSE for the original squat training data versus reconstructed squat data with an increasing number of principal components on the *x* axis.

A statistical model of kinetic output data from the AMS was made for deep squatting and another one for lunging. Figure 2 displays the cumulative variance of modes in the statistical model. The variances of the first three modes in the squat model are illustrated in Figures 3–5. Together they represent 59.73% of the population variance. For the lunge, the first three modes accounts for 66.40% of population variance. More about these modes are detailed in Figures 6–8. In Table 2, the in-sample model accuracy and the specificity median RMSE are described for each model. The boxplots in Figure 9 illustrate the in-sample squat model accuracy for the ascending number of the components included. There is a boxplot for every variable in the model. The median and the interquartile range of the RMSE, when compared to the initial data, decrease as more PCs are included in the reconstructed data, as expected. The model accuracies from the lunge model are quite similar but around one IQR RMSE higher. In contrast, the lunge model is a bit more compact. For calculating the out-of-sample accuracy based on leave-one-out tests, we based on the lunge data because it has the most test data. The results of the model compactness and the statistical findings of the permutation testing related to the number of the principal components used are demonstrated in Table 3.

**TABLE 2:** Validation analyses of the squat and lunge statistical kinetic model. We consider squat and lunge models that capture 80, 90, 95, and 98% of inter-individual population variance.

Validation summary	Squat model				Lunge model			
		
% of inter-variability in the population	80%	90%	95%	98%	80%	90%	95%	98%
Model accuracy RMSE (median ±IQR**) (BW)	0.0149 ±0.0122	0.0107 ±0.0087	0.0075 ±0.0064	0.0054 ±0.0048	0.0248 ±0.0210	0.0162 ±0.0167	0.0132 ±0.0126	0.0082 ±0.0083
Dimensionality*	6	10	14	19	5	9	13	17
Model specificity RMSE (median ±IQR**) (BW)	0.1582±0.0943	0.1581±0.0948	0.1583±0.0946	0.1584±0.0943	0.1291±0.0831	0.1310±0.0815	0.1314±0.0809	0.1320±0.0803

*i.e., the number of modes. **IQR, interquartile range.

**TABLE 3:** Choosing the optimal amount of principal components for the squat kinetic datasets.

PC	Eigenvalue	Percentage	Cumulative	Rank of	Equality
		of variance	variance	roots	of roots
1	204.85	33.80	33.80	0.001	0.001
2	85.11	14.04	47.85	0.001	0.001
3	71.98	11.88	59.73	0.001	0.001
4	58.21	9.61	69.33	0.001	0.001
5	46.81	7.73	77.06	0.001	0.001
6	31.26	5.16	82.22	0.001	0.001
7	18.13	2.99	85.21	0.001*	0.001
8	15.18	2.50	87.71	1	0.001
9	13.82	2.28	89.99	1	0.001
10	9.55	1.58	91.57	1	0.001
11	8.38	1.38	92.95	1	0.001
12	6.37	1.05	94.00	1	0.001
13	5.57	0.92	94.92	1	0.001
14	4.65	0.77	95.69	1	0.005*
15	3.97	0.66	96.35	1	0.078

Type I error probability is set to 0.05. Rank of roots measure suggests that seven principal components (PCs) are statistically significant in meaningfully describing the dataset, corresponding to 85% of data variance, whereas the equality of roots suggests that 14 PCs are to be included (representing 95.7% of data variance). *p < 0.05.

**FIGURE 10:**
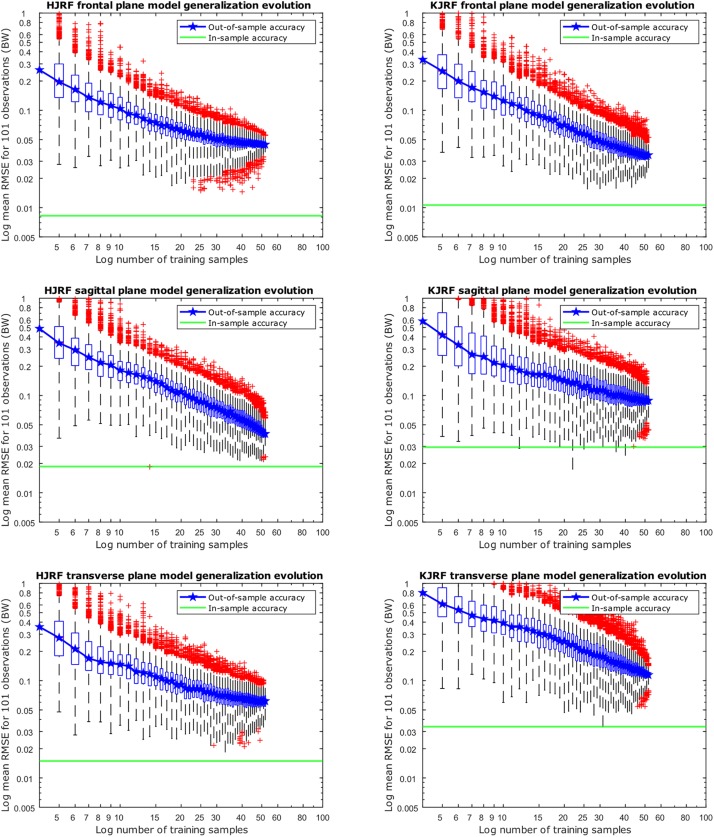
Accuracy evolution of kinetic lunge data with log–log scaling (boxplot with root-mean-square error of the reconstructed data with 95% variance versus the original training data) for different levels of prior knowledge expressed as amounts of training data in a kinetic model. The green horizontal line indicates the in-sample target accuracy.

Regarding Figure 10, for each training data input amount going from 4 to 52, 10,000 models were created, including 95% population variance, to reconstruct an excluded subject. Out-of-sample accuracy RMSE from the reconstructed data versus the original excluded data are given on the *y* axis in box-and-whisker diagrams. The boxplots are log–log-scaled in order to visualize the downward trend of the out-of-sample accuracy. Also plotted is a horizontal line of the in-sample model accuracy of our 95% model. The out-of-sample accuracies are less than 0.1 BW, except for the KJRF in the transverse plane. From 50 test subjects up, the out-of-sample accuracies are clearly stagnating for the HJRF in the frontal and the transverse plane as well as for the KJRF in the sagittal plane.

## Discussion

The validation analysis confirmed that our models have a high degree of compactness and accuracy. Many types of noise are in the higher components. The PCA technique has adequately allowed rejection of the error variance from the model. The meaningful variance is obviously divided over the first 12 or 14 components. This multidimensionality describes the silent features in the data and, eventually, they could be linked to the varying characteristics of the study population. A common source of meaningless variance originates from data alignment. It is impossible to avoid this because we do not want to introduce supplementary noise in the data by aligning them more stringently. Since all subjects have a BMI lower than 25, skin shift errors during movements are limited (Cappozzo et al., 1996).

According to the lunge, the model only describes the closed-chain part of motion for two reasons. First, femoroacetabular impingement and joint reaction forces are more pronounced at higher flexion (Audenaert et al., 2012). Secondly, while creating a model from the onset of the lunge back to the original position, the model would be no longer compact enough because there is too much degree of freedom when moving a leg in the air.

The dominant mode is supposed to describe the overall variance (Jolliffe et al., 2002), as is clearly apparent in the lunge model. In the squat model, the overall variance is limited for the HJRF in the frontal and the sagittal plane. This is due to low hip flexion and rotation moments because the center of gravity will lie almost perfectly between the hip joints (and not the knee joints during squat), in contrast to the lunge case. For this statement, we based on Schwab et al. (2006). They found that, for young adults, the femoral head position appears to be a reliable indicator for the gravity line in the sagittal plane during stance (Schwab et al., 2006).

The second mode of the squat model indicates that a high HJRF component in the transverse plane results in high KJRF components in the frontal and sagittal plane in order to counterbalance the downward force at the hip. The third mode correlates the depth of squatting with the joint reaction force components in the frontal plane. For the lunge, the association of the frontal joint reaction force components is mainly summarized in the second mode. Finally, according to our interpretation, the third mode of the lunge model may take alignment errors into account.

The RMSE for model accuracy are far below 0.05 BW, as opposed to similar studies. The specificity was almost equal for models with 80, 90, 95, and 98% of variance. It questions the relevance of taking the model specificity into account in this setting. According to the generalization evolution, we could conclude that, minimally, 50 samples are enough to provide reliable models at 0.1 BW precision for both squat and lunge motion. Nevertheless, we recommend exceeding this threshold number because the in-sample accuracy is still lower, especially for the squat. Note that gender, age group, BMI group, and race differences are not included here. Therefore, it is very likely that, in more heterogeneous populations like the elderly, 50 samples will be too low to ensure reliable models.

Unfortunately, electromyography data are not collected during this study. This could give information about muscle activation and muscle strength. Motor unit action potentials could be registered non-invasively by using surface electromyography. It has been stated several times that the muscle activation patterns depend on several aspects like training level and osteoarthritis (Benedetti et al., 2003; Knoop et al., 2012; Mei et al., 2017). The integration of electromyography and kinetic data could help to declare aberrant kinetic patterns.

By applying correlation matrix PCA to obtain uncorrelated maximum-variance linear combinations and given that there is only kinetic data with limited scaling differences, some more PCs are required to account for the same amount of covariance compared to classical covariance matrix PCA (Jolliffe et al., 2002; Jolliffe and Cadima, 2016). This makes the selection of PCs in the kinetic data subspace even more crucial to ensure model validation properties like accuracy, compactness, generalization, and specificity, which is the major drawback of PCA (Jolliffe et al., 2002; Peres-Neto et al., 2005; Vasco, 2012). To handle this, there are numerous methods described in the literature, but there is no consensus yet. We objectified our selection strategy based on eigenvalues by considering the validation measures for different cutoffs. On top of that, for the model generalization and specificity abilities, we assume multivariate normal distribution which is seldom true (Vasco, 2012).

The most important limitation of the present work, however, relates to the population under investigation, namely, young male, Belgian adolescents and the unknown extent of which findings can be extrapolated to other populations. Nevertheless, in general terms, we expect our results to be representative by extension for a Western European population.

## Conclusion

We created two models that describe kinetics from both hip and knee joint, contrary to the limited number of studies available with PCA analyses of waveform data considering the knee only (Deluzio and Astephen, 2007; Reid et al., 2010; Galloway et al., 2012). Since all muscles from the knee, except from the M. popliteus (Paulsen and Waschke, 2011), are biarticular and the body should be seen as a whole, a model with the HJRF as well as KJRF is preferable. We proved that such a model for 95% of population variance was compact and very accurate (<0.015 BW). To describe the population at <0.1 BW precision, our small sample size was still sufficient. Using *t*-tests to investigate differences in PC scores in comparing studies will enable the creation of personalized hip and tibial implants with specific weight-bearing properties, resulting in prolonged longevity. In our opinion, this is a very important feature since total knee arthroplasty and total hip arthroplasty are increasingly utilized to treat more physically active patients.

## Data Availability Statement

The datasets generated for this study are available on request to the corresponding author.

## Ethics Statement

The studies involving human participants were reviewed and approved by the Commission for Medical Ethics, UZ Gent. The patients/participants provided their written informed consent to participate in this study.

## Author Contributions

All authors listed have made a substantial, direct and intellectual contribution to the work, and approved it for publication.

## Conflict of Interest

PG is an employee of AnyBody Technology. No financial benefits have been received or will be received from any commercial party related directly or indirectly to the subject of this article. The remaining authors declare that the research was conducted in the absence of any commercial or financial relationships that could be construed as a potential conflict of interest.
